# 4536 Identification of the most salient risk factors of preterm birth in the US using geospatial mapping

**DOI:** 10.1017/cts.2020.131

**Published:** 2020-07-29

**Authors:** Alexander J Layden, Janet Catov

**Affiliations:** 1 University of Pittsburgh

## Abstract

OBJECTIVES/GOALS: Preterm birth is the most common birth complication in the United States. To date, there are no effective public health strategies to reduce the burden of prematurity. Using geospatial information system (GIS) mapping, we identified the most salient risk factors of preterm birth across US counties targetable for future interventions. METHODS/STUDY POPULATION: Risk factors of preterm birth were identified from the perinatal health nonprofit organization, March of Dimes, and included factors such as obesity, smoking, insurance coverage and poverty. US 2013 county-level data on sociodemographic characteristics, behavioral risk factors and preterm birth were extracted and combined from the American Census, Center for Disease Control, and US Health Resources and Services Administration. Spatial autocorrelation and multivariate spatial regression were used to determine the risk factors most strongly associated with preterm birth. These models were adjusted for race, given well-documented race disparities for preterm birth. As a case-study comparison, we mapped risk factors in the two states with the highest and lowest proportion of preterm births in 2013. RESULTS/ANTICIPATED RESULTS: In our preliminary analysis, obesity was the factor most strongly associated with preterm birth (ß = 7.32, SE: 1.13, p<0.001) at the US county-level. Surprisingly, smoking was not found to be significantly associated with preterm birth. In 2013, Vermont had the lowest prevalence of preterm birth at 7.6% and Mississippi had the highest prevalence of preterm birth at 13.1%. Health insurance coverage and obesity were the two risk factors that differed between Vermont and Mississippi. The median proportion of uninsured individuals in Mississippi counties was four times higher than that of Vermont counties (26.3% vs 10.9%, p<0.01). Similarly, the median obesity prevalence in Mississippi counties was significantly higher than the median obesity prevalence in Vermont counties (38.8% vs. 25.2%). DISCUSSION/SIGNIFICANCE OF IMPACT: Public health efforts aimed at reducing obesity and increasing health insurance coverage may have the greatest impact at addressing the US burden of preterm birth. Further, geospatial mapping is a powerful analytic tool to identify regions in the US where preterm birth interventions would be most beneficial.

